# Protective effect of quercetin on pulmonary dysfunction in streptozotocin-induced diabetic rats via inhibition of NLRP3 signaling pathway

**DOI:** 10.1007/s11356-023-25254-8

**Published:** 2023-01-17

**Authors:** Noha Osama El-Shaer, Ahmed Medhat Hegazy, Marwa H. Muhammad

**Affiliations:** 1 Department of Physiology, Faculty of Medicine, Benha University, Benha, Egypt; 2 Department of Forensic Medicine and Toxicology, Faculty of Veterinary Medicine, Benha University, Moshtohor, Toukh, 13736 Qalyubia Egypt

**Keywords:** Arterial blood gases, Diabetic lung, NLRP3 inflammasome, Oxidative stress, Pyroptosis, Quercetin

## Abstract

Diabetes mellitus (DM) is a dysmetabolic disease characterized by chronic hyperglycemia. In the developed countries, DM is the commonest life style disease that affects both old and young age. Nod-like receptor protein-3 (NLRP3)-mediated pyroptosis may in fact aid in the development of diabetic complications. Quercetin is a natural flavonoid, can be present in natural foods and plants. Many studies have reported the antioxidant role of quercetin on different tissues, but its effects on NLRP3-mediated pyroptosis in diabetic lung are unclear. The current study aimed to assess quercetin’s protective effects on lung function, oxidative stress, and NLRP3-mediated pyroptosis in Wister rats exposed to streptozotocin (STZ)-induced DM. Forty male Wister rats were randomly allocated into four equal groups. The groups of rats were as follows: group 1 (G1) was kept under normal control conditions; G2 was injected I/P quercetin at a dose of 30 mg/kg b.wt., daily for 30 days; G3 and G4 were injected with a single dose of streptozotocin (STZ) 50 mg/kg b.wt. I/P to induce DM. After 72-h post diabetes induction, the rats of G4 were treated with quercetin as a manner in the second group. The results showed that quercetin ameliorates the pulmonary dysfunctions caused by DM through restoring the levels of glucose, insulin, and arterial blood gases, as well as the oxidative markers. Also, NLRP3-pyroptosis-mediated IL1β was inhibited. Quercetin also reduces the effect of DM on the lung by decreasing the pathological changes in the lung. In conclusion, NLRP3 inflammasome-induced pyroptosis may aggravate lung injury in diabetic rats. Quercetin has the potential to ameliorate diabetes induced pulmonary dysfunction by targeting NLRP3.

## Introduction

Diabetes mellitus (DM)—a dysmetabolic disease—is predicted to affect approximately 642 million people worldwide by 2040 (Zheng et al. 2018). It is characterized by chronic hyperglycemia. It affects the heart, brain, liver, kidney, skeletal muscle, and eyes (Talakatta et al. 2018). Lung is also one of the target organs for diabetes (Zheng et al. 2017). DM causes gradual loss of function in these vital organs. Diabetes has been found to affect pneumocytes (type II). The affected pneumocytes reduce surfactant formation and secretion leading to reduce lung volume and decrease elasticity (Südy et al. 2020). Even with these findings, there are still few studies which elaborate on the pathophysiological mechanisms by which DM affects pulmonary function.

Pyroptosis, is associated with inflammatory response and can be defined as intrinsic cell death mechanism. It is a double-edged sword for innate immunity; it protects multicellular organisms but excessive activation of pyroptosis may lead to chronic inflammation (Lu et al. 2020). There are multi-protein complexes known as inflammasomes associated with the innate immune system of pyroptosis (Zhu et al. 2017). The NLRP3 (Nod-like receptor pyrin domain-containing 3) inflammasome is one of them. The ROS (reactive oxygen species) activate NLRP3 in β cells, speeding up caspase-1-dependent IL-1β (interleukin-1β) production in a hyperglycemic environment. The response causes abnormal insulin release from beta cells, ultimately leading to the development of DM. Thus, NLRP3 inflammasome activation may be correlated with pyroptosis in diabetes (Yu et al. 2020). Gasdermin D (GSDMD), is required for pyroptosis. It is a physiological substrate included in the canonical inflammasome pathway. GSDMD initiating maturation of IL-1β release and then forms cytotoxic pores within the cell membrane. Simultaneously, it was found that it was cleaved by both pro- and mature caspase-1 (Cas-1) and recruited to the NLRP3 inflammasome (He et al. 2015).

Quercetin, a natural flavonoid, can be found in natural foods and plants including berries, cilantro, dill, apples, and onions. Quercetin supplements have been found to normalize blood glucose level through both its anti-diabetic and antioxidant properties (Refat et al. 2021). In diabetic rat models, it exerted protective effects via inhibiting the activation of NLRP3 in cardiac tissue (Yao et al. 2021), in retinopathy (Chai et al. 2021), and in encephalopathy (Hu et al. 2020). Therefore, the goal of the current investigations was to assess the possible protective and immunomodulatory effects of quercetin on the lung functions of Wister rats subjected to streptozotocin (STZ) caused DM.

## Materials and methods

### Chemicals

Streptozotocin (STZ) (Cat#18,883–66-4) and Quercetin (Cat#849,061–97-8) were purchased from Aldrich Company, USA.

### Experimental animals

Forty male adult Wister rats, 6 weeks old and weighing between 125 and 145 g, were obtained from the Animal House, National Research Centre, Egypt (Dokky, Giza, Egypt). The rats were kept under hygienic conditions and given a free clean water and balanced diet ad libitum. The rats were kept at ambient temperature (23 ± 3 °C) and exposed to a natural daily 12-h dark/light cycle. At the end of the experiment, and after the collection of samples, rats were disposed of by using the incinerator.

### Induction of diabetes mellitus

Streptozotocin was prepared in 0.1 M citrate buffer at pH 4.5. STZ was injected as a single dose of 50 mg/kg b.wt. I/P to induce DM according to the method of Roslan et al. (2017). The rats drank overnight, a solution containing 5% glucose to avoid hypoglycemia. Diabetes mellitus was verified 72 h later, by measuring the blood glucose levels (after an overnight fasting). A glucose level of 250 mg/dl or more was considered as DM (Hussien and Shoman 2015).

### Experimental design

The experimental rats were allocated randomly into equal four groups of ten rats each. The group 1 (G1) was preserved as a normal control, and the group 2 (G2) was administered I/P quercetin at a dose of 30 mg/kg b.wt. (Refat et al. 2021) that dissolved in 20% glycerol in physiological saline daily for 30 days. The group 3 (G3) (diabetic group) and the group 4 (G4) (diabetic then treated) were injected with a single dose of streptozotocin (STZ) 50 mg/kg b.wt. I/P to induce DM. After 72-h post diabetes induction, the rats of G4 were treated with quercetin as a manner in the G2. At the end of the experiment, and after fasting overnight, the experimental rats were euthanized, then blood samples from the abdominal aorta and lung tissue were collected for estimation of the glucose, insulin level, arterial blood gases, oxidative/antioxidative markers, pulmonary proteins, gene expression, and the histopathological changes in lungs.

### Preparation of lung homogenate

Lung tissue homogenate was performed according to Hegazy et al. (2020). The collected supernatant was used for determination of the oxidant/antioxidant markers [lipid peroxidation by-products (MDA) level, superoxide dismutase (SOD) activity, and reduced glutathione (GSH) concentration], pulmonary proteins [NLRP3, Cas-1, GSDMD, IL-1β, and surfactant protein type B (SP-B)], and the total protein.

### Assay methods

The fasting serum glucose level was performed according to Trinder (1969). Fasting serum insulin level was estimated by the method of Groen et al. (1952).

### Assessing lung function

The blood samples were drawn from the abdominal aorta on heparin. Arterial blood gases: PaO_2_ [the partial pressure of oxygen in arterial blood (millimeter of Mercury; mmHg)], PaCO_2_ [the partial pressure of carbon dioxide in arterial blood (mmHg)], and HCO_3_ [the concentration of bicarbonate in arterial blood (mmol/l)], and pH [the negative log of the hydrogen ion concentration (acid–base balance of the blood)] were performed according to Castro et al. (2021) using the blood gas analyzer (GEM Premier 3500 PAK).

### Oxidant/antioxidant markers in lung homogenate

The MDA (Garcia et al. 2005), SOD activity (Flohe 1984), GSH content (Ellman 1959), and total proteins content (Lowry et al. 1951) were determined in lung tissue homogenate.

### Protein markers in lung homogenate

Anti-NLRP3Ab, anti-Cas-1Ab, anti-GSDMDAb, anti-IL-1βAb, and anti-SP-B in lung tissue homogenate were detected by a commercially available, highly specific ELISA kits (NLRP3 kit, Aviva Systems Biology, San Diego, USA), (caspase-1, BioVision, Milpitas, USA), (GSDMD, Biomatik, Ontario, Canada), (IL-1β, Cloud-Clone Corp., Katy, USA), and (SP-B, Novus, Ontario, Canada). The microtiter plates coated with the specific protein to find the specific anti-protein Ab or with control proteins to find nonspecific interactions. The assay was performed according to the manufacturer’s instructions. Absorbance was measured using Labsystems Multiscan MS ELISA reader (DASIT).

### Pulmonary mRNA gene expression

The mRNA gene expression of pulmonary NLRP3, Cas-1, GSDMD, and IL-1β genes were determined by real-time polymerase chain reaction (PCR). Primer sets of NLRP3, Cas-1, GSDMD, IL-1β, and β–actin were used (Table 1) as previously described by Farid et al. (2010). A 7300 real-time PCR system was used to conduct thermal cycling and fluorescence detection (Applied Biosystems, Foster City, CA, USA). Changes in gene expression were determined from the obtained cycle threshold (Ct) values from real-time PCR equipment to a reference (housekeeping) gene (β–actin) (Schmittgen and Livak 2008).

**Table 1:** The primer sets of the assessed genes

Genes	Forward primer (sense)	Reverse primer (antisense)
NLRP3	5′- CAGACCTCCAAGACCACGACTG-3′	5′- ATGTCCTGGGAAGAGGTAGAAACG-3′
Caspase-1	5′-TGCCTGGTCTTGTGACTTGGAG-3′	5′- ATGTCCTGGGAAGAGGTAGAAACG-3′
GSDMD	5′-CCAACATCTCAGGGCCCCAT-3′	5′- TGGCAAGTTTCTGCCCTGGA-3′
IL-1β	5′-TTGAGTCTGCACAGTTCCCC-3′	5′-TCCTGG GGAAGGCATTAGGA-3′
β–actin	5′-GGCTGTATTCCCCTCCATCG-3′	5′-CCAGTTGGTAACAATGCCATGT-3′

### Histopathological investigations

Lung specimens were immediately taken from each rat and fixed for 24 h in 10% buffered neutral formalin. The specimens were rinsed in running water, dehydrated in various grades of ethyl alcohol, clarified in xylol, embedded in paraffin, blocked, and sectioned into 5 µm in thickness, then microscopically examined after being stained with hematoxylin and eosin (H&E) (Bancroft et al. 1994). Five random fields from each slide were analyzed. Slides were photographed using Olympus® digital camera installed on Olympus® microscope with 0.5 × photo adaptor, using 20 × objective and saved as TIFF. The result images were analyzed on Intel® Core I7®-based computer using VideoTest Morphology® software (Russia) with a specific built-in routine for area measurement. Empty areas were selected and subjected to area measurement routine. Results were exported to Excel sheet expressed as % area of alveolar cavity in relation to total field area. Data were used to estimate the degree of emphysematous sac, distorted bronchi, and thick alveolar wall. The higher values indicate normal tissue architecture.

### Statistical analysis

The data was expressed as the mean ± SD (standard deviation). One-way analysis of variance (ANOVA) was used to assess the significance of differences between groups of treatment followed by Duncan’s multiple range test (Kinnear and Gray 2006) using SPSS version 20 computer program (SPSS Inc., Chicago, USA). The value of *p* < 0.05 is considered significant.

## Results

During the experimental period, no mortalities were recorded in all groups of treatment.

### Serum glucose and insulin

The mean and standard deviation upsides of blood glucose and insulin level of the different gatherings are portrayed in Table 2. The fasting blood glucose level was markedly increased in the diabetic rats (G3) compared to both normal control and quercetin-treated groups after 30 days. However, diabetic rats treated with quercetin recorded a marked decrease fasting blood glucose level after 30 days of treatment in comparison with the diabetic group. Insulin level also was markedly decreased in the diabetic group compared to both control and quercetin-treated groups. However, diabetic rats treated with quercetin showed marked increases in insulin level after 30 days of treatment when compared with the diabetic group.

**Table 2:** Effect of quercetin against streptozotocin (STZ) on blood glucose level and insulin in various gatherings of the experiment after 30 days of treatment (mean ± standard deviation), (*n* = 10)

Groups	Control	Quercetin	STZ	Quercetin and STZ
Glucose (mg/dl)	86.318 ± 4.471^c^	89.228 ± 3.392^c^	328.021 ± 15.080^a^	144.789 ± 6.035^b^
Insulin (µIU/ml)	26.707 ± 3.073^a^	27.427 ± 2.809^a^	3.878 ± 0.558^c^	16.696 ± 1.835^b^

Means with different superscripts in the same row are significantly different at *p* < 0.05

### Changes in arterial blood gases

The mean and standard deviation upsides of arterial blood gases of the different gatherings are portrayed in Table 3. The PaO_2_, pH, and HCO_3_ were markedly decreased in the diabetic rats compared to normal control and quercetin-treated groups after 30 days of treatment. However, diabetic group treated with quercetin revealed markedly increased PaO_2_, pH, and HCO_3_ after 30 days of treatment when compared with the diabetic group. The PaCO_2_ was significantly increased in the diabetic group in comparison with both normal control and quercetin-treated groups after 30 days of treatment. However, diabetic rats treated with quercetin showed marked decreased in PaCO_2_ level after 30 days of treatment compared to the diabetic group.

**Table 3:** Effect of quercetin against streptozotocin (STZ) on arterial blood gases in various gatherings of the experiment after 30 days of treatment (mean ± standard deviation), (*n* = 10)

Groups	Control	Quercetin	STZ	Quercetin and STZ
PaO_2_ (mmHg)	91.405 ± 0.962^a^	91.687 ± 1.502^a^	73.378 ± 2.028^c^	81.953 ± 2.268^b^
PaCO_2_ (mmHg)	39.206 ± 1.337^c^	39.184 ± 2.054^c^	51.539 ± 1.489^a^	42.965 ± 1.535^b^
pH	7.363 ± 0.011^a^	7.360 ± 0.012^a^	7.214 ± 0.006^c^	7.272 ± 0.030^b^
HCO_3_ (mmol/l)	24.372 ± 0.996^a^	24.778 ± 1.147^a^	20.575 ± 0.936^c^	23.035 ± 0.557^b^

Means with different superscripts in the same row are significantly different at *p* < 0.05

### Changes in the lung oxidant/antioxidant markers

The mean and standard deviation values of oxidant/antioxidant markers of the various groups are depicted in Table 4. In the pulmonary tissue, the level of MDA was markedly increased in the diabetic rats than normal control and quercetin-treated groups. Co-treatment of the diabetic group with quercetin showed significant decreases in MDA level. The values of SOD and GSH in the pulmonary tissue of the diabetic rats were markedly decreased than the normal control and quercetin-treated groups. On the other hand, co-treatment of the diabetic rats with quercetin showed significant increases in the values of SOD activity and GSH.

**Table 4:** Effect of quercetin against streptozotocin (STZ) on oxidative/antioxidative markers in the lung homogenates of various gatherings of the experiment after 30 days of treatment (mean ± standard deviation), (*n* = 10)

Groups	Control	Quercetin	STZ	Quercetin and STZ
MDA (nmol/mg protein)	39.757 ± 1.247^c^	40.776 ± 1.521^c^	112.668 ± 5.908^a^	76.787 ± 2.743^b^
SOD (U/mg protein)	0.255 ± 0.006^a^	0.248 ± 0.016^a^	0.121 ± 0.006^c^	0.189 ± 0.037^b^
GSH (µmol/mg protein)	14.755 ± 1.107^a^	13.566 ± 0.739^a^	5.549 ± 0.695^c^	8.965 ± 0.337^b^

Means with different superscripts in the same row are significantly different at *p* < 0.05

### Changes in pulmonary protein markers

The mean and standard deviation values of pulmonary protein markers of the various groups are depicted in Table 5. There were marked increase in the NLRP3, Cas-1, GSDMD, and IL-1β levels and significant decrease in SP-B protein marker in lung tissue in the diabetic group compared to both normal control and quercetin-treated groups. Co-treatment of the diabetic group with quercetin showed marked decrease in the levels of NLRP3, Cas-1, GSDMD, and IL-1β but marked increases in SP-B compared with the diabetic group after 30 days of the experiment.

**Table 5:** Effect of quercetin against streptozotocin (STZ) on pulmonary protein markers in the lung homogenates of various gatherings of the experiment after 30 days of treatment (mean ± standard deviation), (*n* = 10)

Groups	Control	Quercetin	STZ	Quercetin and STZ
NLRP3 (ng/mg protein)	0.555 ± 0.089^c^	0.515 ± 0.058^c^	3.059 ± 0.418^a^	1.205 ± 0.156^b^
Caspase-1 (pg/mg protein)	73.707 ± 4.366^c^	71.821 ± 3.609^c^	288.372 ± 6.604^a^	113.293 ± 5.557^b^
GSDMD (ng/mg protein)	0.563 ± 0.204^c^	0.523 ± 0.085^c^	2.739 ± 0.172^a^	1.192 ± 0.133^b^
IL-1β (pg/mg protein)	40.427 ± 1.987^c^	40.527 ± 1.499^c^	154.489 ± 7.179^a^	61.884 ± 2.149^b^
SP-B (ng/mg protein)	31.055 ± 0.558^a^	32.032 ± 1.523^a^	9.415 ± 0.906^c^	25.366 ± 1.453^b^

Means with different superscripts in the same row are significantly different at *p* < 0.05

### NLRP3, Cas-1, GSDMD, and IL-1β mRNA expression in pulmonary tissue

**Fig. 1:**
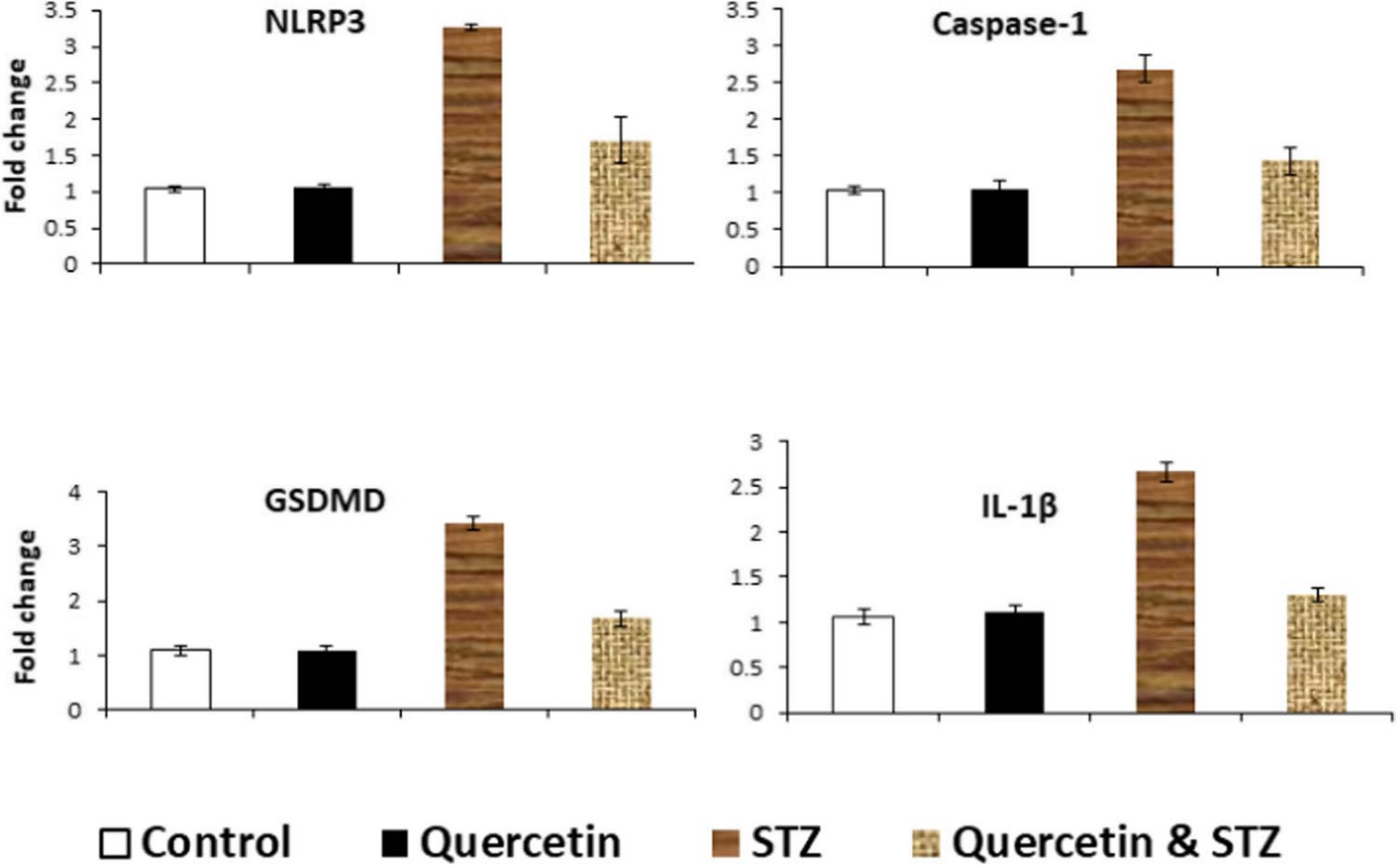
mRNA expression of pulmonary NLRP3, caspase-1, GSDMD, and IL-1β. Total RNA was prepared from pulmonary tissues of rats treated with quercetin, STZ, quercetin against STZ, and control on 30 days after treatments. Real-time PCR was evaluated the expression levels. *p* < 0.05 compared with control values. Bars represent means ± S.D.M. (*n* = 10)

As displayed in Fig. 1, the expression of lung NLRP3, Cas-1, GSDMD, and IL-1β mRNA genes in the control, diabetic, quercetin, and diabetic-quercetin-treated groups after 30 days of treatment. The diabetic rats induced a marked upregulation in lung NLRP3, Cas-1, GSDMD, and IL-1β mRNA. However, treatment of diabetic rats with quercetin induced a marked downregulation of these genes.

### Histopathological assessment of lung tissue

**Fig. 2:**
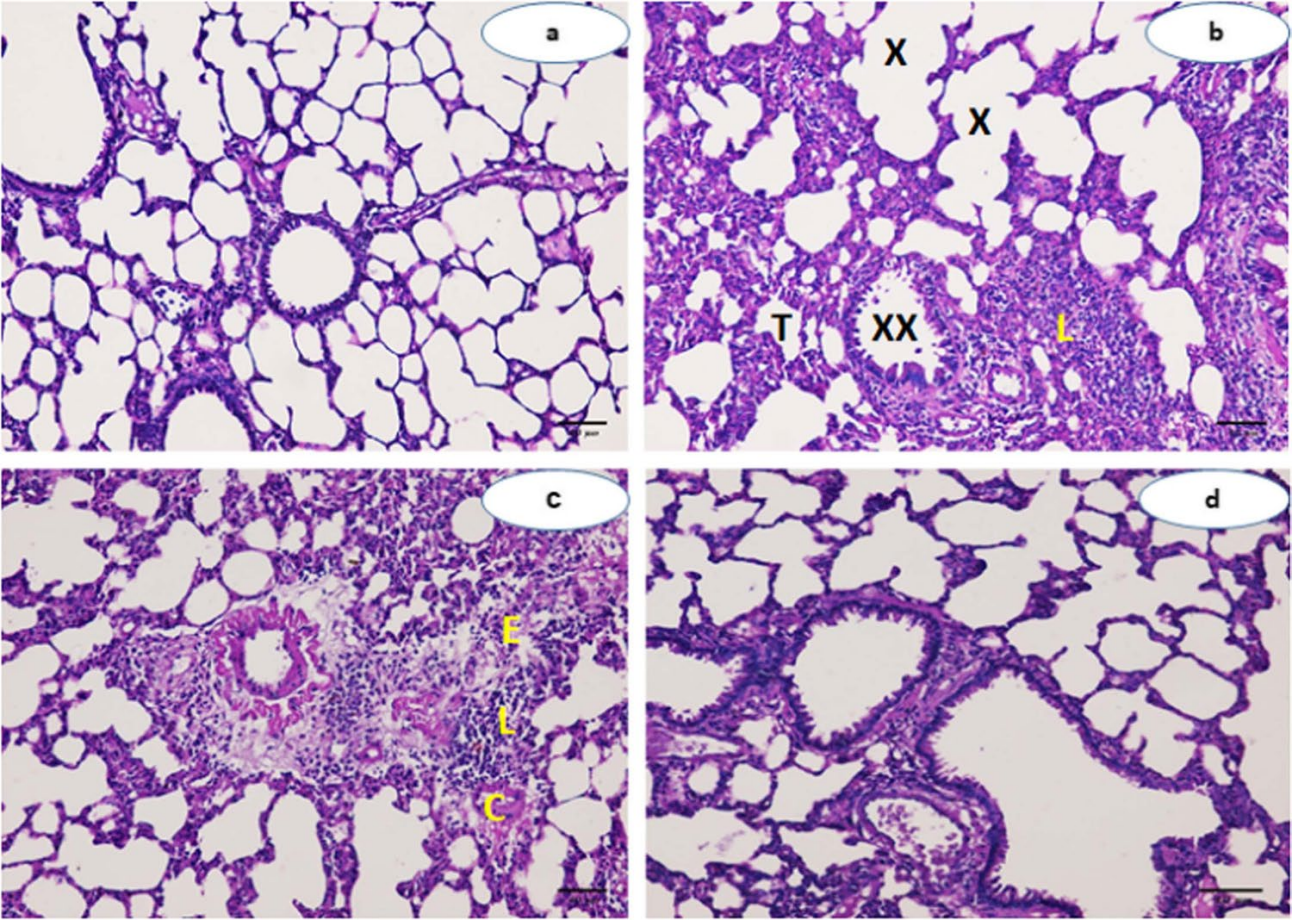
Photomicrograph from lung of experimental rats. (**a**) Normal control rat (G1) and rat treated with quercetin (G2), the lung showing a normal histological structure with no abnormalities. (**b**, **c**) Diabetic rats (G3) induced by streptozotocin. (**b**) the lung showing emphysematous sac (X), distorted bronchi (XX), thick alveolar wall (T), and massive lymphocytic cell infiltration (L), and (c) the lung showing significant congestion (C) and interstitial edema (E). (**d**) Diabetic rat then treated (G4), induced by streptozotocin then treated with quercetin, showing normal alveolar sac with mild cellular infiltration. (H&E, × 200) (Scale bar represents 256 μm)

**Fig. 3:**
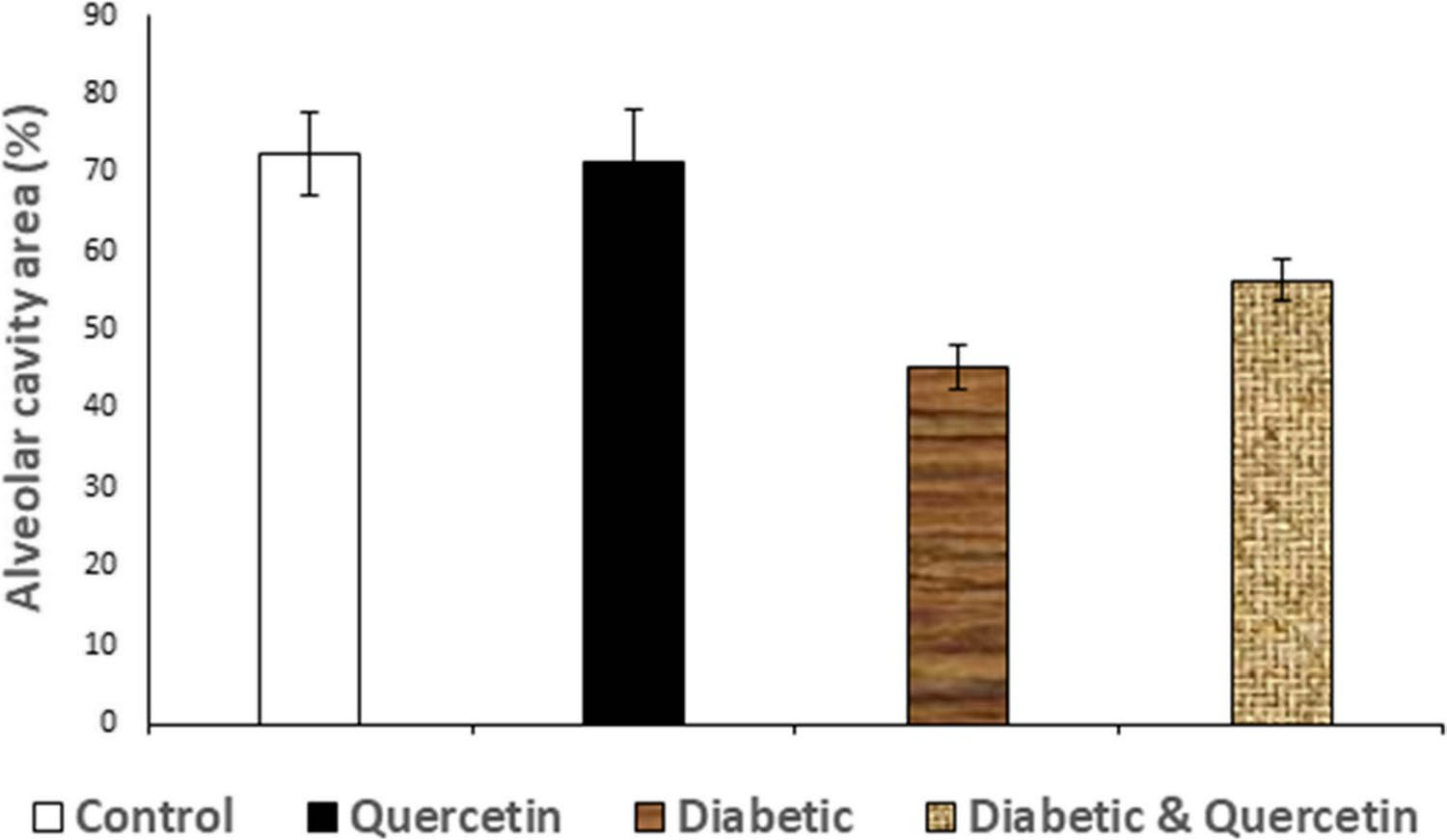
Digital morphometric study of lung of experimental rats. Lung empty areas of rats treated with quercetin, STZ, quercetin against STZ, and control on 30 days after treatments were selected and subjected to area measurement routine. Results were expressed as alveolar cavity area (%) in relation to total field area. Data were used to estimate the degree of emphysematous sac, distorted bronchi, and thick alveolar wall. The higher values indicate normal tissue architecture. *p* < 0.05 compared with different experimental groups. Bars represent means ± S.D.M. (*n* = 10)

Examination of lung sections revealed that diabetic rats showed emphysematous sac (X), distorted bronchi (XX), thick alveolar wall (T), massive lymphocytic cell infiltration (L), as well as significant congestion (C) with appearance of interstitial edema (E) (Fig. 2b, c). The alveolar cavity area was markedly decreased in the diabetic rats (G3) compared to both normal control and quercetin-treated groups after 30 days (Fig. 3). Co-treatment of diabetic rats with quercetin showed normal alveolar sac with mild cellular infiltration (Fig. 2d). The alveolar cavity area was markedly increased when diabetic group treated with quercetin (G4) in comparison with diabetic group (Fig. 3). The pulmonary tissues of rats that administered quercetin appeared to be normal (Fig. 2a).

## Discussion

This study was designated to research the adequacy of quercetin on keeping up with the lung functions of Wister rats administered STZ inducing diabetes mellitus. DM is a complex syndrome characterized by hyperglycemia, oxidative stress, and inflammation that can prompt loss of visual, renal, and neurologic capabilities (Atta et al. 2020). The significant reason for death in diabetic patients is glucotoxicity-prompted complications (Zheng et al. 2017). In this experiment, there were no evident clinical indications of harmfulness of STZ at the dose of 50 mg/kg b.wt., which revealed no signs of stress was seen on the rats.

This study revealed that fasting blood glucose level significantly increased while serum insulin level markedly decreased in the G3 which indicates DM. The elevated level of blood glucose and decrease level of insulin may have been due to STZ is like glucose to be moved into the beta cells of the pancreatic islets by the glucose transport protein, yet is not perceived by the other glucose carriers. This clears up its overall harmfulness for beta cells of the pancreatic islets, since these cells have relatively high levels of glucose transporter 2GLUT2 (Wang and Gleichmann 1998). A similar increase in blood glucose level caused by STZ has been reported in a previous study (Roslan et al. 2017). STZ is a glucosamine-nitrosourea compound that is harmful to cells by making harm the DNA. Damage to DNA triggers the start of poly ADP-ribosylation, which is probably more crucial for the development of diabetes than DNA damage itself (Szkudelski 2001). STZ decreases nicotinamide-adenine dinucleotide (NAD) in beta cells of pancreas, which are more sensitive than other cells to STZ challenge, and causes degeneration of the beta cells in the islet of Langerhans and intermediates induction of diabetes within 3 days (Elsner et al. 2000). A significant decrease in the blood glucose level and increase in the insulin level were recorded when diabetic rats treated with quercetin. This outcome might have been due to the pancreatoprotective activity of the quercetin. A previous report revealed that quercetin was able to decrease destruction of β-cells of pancreas caused by STZ (Roslan et al. 2017). Quercetin could work in concordant with nicotinamide to give assurance to the pancreas. It safeguards the pancreas against oxidative stress prompted impedance in insulin secretion. It has also been shown to enhance insulin secretion and alleviate degeneration of the pancreas in diabetic rats (Bhattacharya et al. 2014).

In the current study, arterial blood gas analysis showed a marked increase in the arterial blood PaCO_2_ and a significant decrease in arterial blood PaO_2_, pH, and HCO_3_ levels observed in the diabetic group at 30 days of receiving STZ, that indicates respiratory acidosis. The elevated level of PaCO_2_ and decrease level of PaO_2_, pH, and HCO_3_ may have been due to the inflammation associated with DM, which contributes to the alveolar-capillary dysfunction. Also the increase in the basement membrane thickness may also be one of the factors responsible for this alteration (Chen et al. 2018). The PaCO_2_ level was markedly decreased, while the levels of PaO_2_, pH, and HCO_3_ were significantly increased when diabetic rats treated with quercetin. Quercetin enhance the formation of surfactant protein B (SP-B) within the alveoli that restore their functions. The surfactant protein B is an important component of surfactant, which is required for normal lung functionality (Tripathi et al. 2021). Changes in the composition or quantity of SP-B may result in alveolar edema, respiratory distress, and subsequent respiratory failure (Tripathi et al. 2021). These findings confirmed with the improvement in histopathological pictures of the lungs of quercetin-treated rats when compared with the lungs of diabetic rats, which showed emphysematous sac, distorted bronchi, thick alveolar wall, massive lymphocytic cell infiltration, as well as significant congestion with appearance of interstitial edema were detected, in addition, markedly decreased in the alveolar cavity area. These findings coincided with those of a previous research (Dubo et al. 2019), while diabetic rats treated with quercetin resulted in normal alveolar sac with mild cellular infiltration and markedly increased in the alveolar cavity area. These findings coincided with Refat et al. (2021) who reported similar findings***.***

The current study revealed that MDA value was a marked increased while SOD and GSH values were significantly decreased in diabetic group after 30 days of receiving STZ which indicated oxidative stress. The elevated level of MDA and decrease level of SOD and GSH may have been due to STZ inducing hyperglycemia and causes tissue damage through increasing the lipid peroxidation by-products and decrease the level of antioxidants (Aggul et al. 2022). On the other side, there was a significant decrease in the level of MDA and significant increase in the level of SOD and GSH when diabetic rats treated with quercetin. Quercetin has antioxidant potential and these findings agree with Feng et al. (2022) who observed the antioxidant effects of quercetin in diabetic nephropathy.

To elucidate more about the role of quercetin in diabetic lung, we studied the role of pyroptosis. The present study revealed that there was a marked increase in the NLRP3, Cas-1, GSDMD, and IL-1β protein markers in pulmonary tissue which confirmed by significant upregulation in lung NLRP3, Cas-1, GSDMD, and IL-1β mRNA observed in the diabetic group at 30 days of receiving STZ, which indicates pyroptosis-mediated cell death. Pyroptosis is considered a type of programmed-inflammatory cell death that elaborates in the pathogenesis of diabetic lung injury (Wang et al. 2021). Hyperglycemia induced ROS generation which leads to the activation of NLRP3 inflammasome, thereby inducing lung injury (Tian et al. 2021). The activated NLRP3 proteins polymerize and combine with the apoptosis-associated speck-like protein containing a CARD, i.e., caspase activation and recruitment domain (ASC adaptor), which subsequently induces the translocation and activation of pro-caspase-1 to Cas-1 (Wang et al. 2018). Cas-1 is the premise of IL-1β production which is released to extracellular domain and triggers inflammatory reactions (Han et al. 2021)***.*** Cas-1 can cleave a full-length GSDMD protein to generate the GSDMD N-terminus, which subsequently forms large oligomeric pores within the plasma membrane, thereby allowing the release of IL-1β and subsequent inflammatory response and cell lysis (Aluganti Narasimhulu and Singla 2021). The present findings related to pyroptosis and pathogenesis of diabetes and its complications agree with Li et al. (2020) who showed its role in diabetes-associated depression. On the other side, diabetic group treated with quercetin showed significant decreases in the levels of NLRP3, Cas-1, GSDMD, and IL-1β protein markers in lung tissue and significant downregulation in lung NLRP3, Cas-1, GSDMD, and IL-1β mRNA gene expression. These findings attributed to anti-inflammatory effects of quercetin via inhibiting NLRP3 inflammasome. These findings agree with Chai et al. (2021) who explained the potential protective mechanisms of quercetin in diabetic retinopathy. Saeedi-Boroujeni and Mahmoudian-Sani (2021) showed that, the antioxidant properties of quercetin can inhibit NLRP3 inflammasome activity via thioredoxin-interacting protein (TXNIP) inhibition and increasing the expression of Sirtuin 1 (SIRT1) thus preventing the exacerbation of inflammation. Moreover, Zhang et al. (2021) confirmed that quercetin downregulated the inflammatory response and pyroptosis through toll like receptor 4 TLR4/NF-κB/NLRP3 pathway. Li et al. (2021) suggested that quercetin may inhibit interleukin 1 receptor-associated kinase-1 IRAK1/NLRP3 signaling pathway, thereby stopping IL-1β-induced chondrocyte injuries in rats.

## Conclusions

Pulmonary injury as one of the diabetic complications is mediated by pyroptosis which is induced by triggering NLRP3–Cas-1–GSDMD–IL-1β pathway. Diabetes-induced lung dysfunction was effectively ameliorated with the use of quercetin via inactivating NLRP3-mediated pyroptosis, which may be a new strategy to mitigate diabetic complications. This study has not only elaborated on the underlying pathology of diabetes-induced lung injury, but also provided a background for possible therapeutics that may prove beneficial.

### Limitation of the study

Further research is required to compare the present study with clinically used compounds.

## Data Availability

Upon a request, the corresponding author will provide the data that support the findings of this study.
